# The effects of artificial aging on high translucent zirconia

**DOI:** 10.1080/26415275.2019.1684201

**Published:** 2019-11-07

**Authors:** Wen Kou, Klas Garbrielsson, Adrian Borhani, Markus Carlborg, Margareta Molin Thorén

**Affiliations:** a Department of Odontology, Umeå University, Umeå, Sweden;; b Department of Applied Physics and Electronics, Umeå University, Umeå, Sweden

**Keywords:** High translucent zirconia, 5Y-PSZ, aging, biaxial flexural strength, transparency, surface roughness

## Abstract

Zirconia is known for its high strength but lacking translucency. Recently, a new type of high translucent zirconia, 5 mol% yttria partially stabilized zirconia (5Y-PSZ), with a larger fraction of cubic zirconia phase has become commercially available. However, the resistance to aging of these commercially available zirconia materials is not yet fully established.

**Purpose: **The aim of the present study was to analyze the effects of artificial aging on surface roughness, transparency, phase transformation and biaxial flexural strength of two 5Y-PSZ products, DD cubeX^2^ and Prettau Anterior.

**Materials and methods: **The artificial aging was performed in an autoclave under 2 bars of pressure at 134 °C for 10 hours, which is estimated to correspond to 30–40 years *in vivo*. Artificial aging for 10 hours had no significant effect on surface roughness, transparency, or phase transformation for either of the tested materials.

**Results:** DD cubeX^2^ had higher mean flexural strength than Prettau Anterior both before and after artificial aging for 10 hours (*p* < .05). DD cubeX^2^ showed, however, a significant reduction in flexural strength after artificial aging (*p* < .05), whereas Prettau Anterior showed a slight increase in flexural strength after artificial aging but not at a significant level.

**Conclusion:** Within the limitation of the present study, both DD cubeX^2^ and Prettau Anterior seems to be relatively resistant to aging. However, a wider range of measured flexural strength indicated that Prettau Anterior probably is a less stable material than DD cubeX^2^, which also means that the flexural strength of DD cubeX^2^ could be more predictable.

## Introduction

Recently 5 mol% yttria partially stabilized zirconia (5Y-PSZ) has become commercially available with claims of a transparency like lithium disilicate. This material is often referred to as high translucent zirconia. Doping the material with 5 mol% yttria increases the amount of cubic crystals in the material to approximately 50% with the benefit of an increased transparency because of cubic zirconia’s isotropic refractive index property [1,2]. An increased translucency comes often at the cost of strength and toughness, as cubic zirconia does not undergo the stress-induced transformation [2]. When environmental stresses transform the metastable tetragonal zirconia to monoclinic zirconia it is referred to as *aging* [3,4]. One hour of accelerated aging in an autoclave at 134 °C with a pressure of 2 bars, theoretically amounts to 3–4 years *in vivo* at 37 °C [5]. The effects of aging can be measured by analyzing the amount of phase transformations from tetragonal to monoclinic in the material. An X-ray diffraction analysis measures the fractions of a phase in the material by radiating the material with a beam of X-rays and measuring the scattering caused by atoms in the crystal structure using a diffractometer [6]. Changes in mechanical properties such as flexural strength, fracture toughness and Vickers hardness (HV) are common after aging [7].

High translucent zirconia is a new material of which several products are commercially available but its resistance to aging is not yet fully established. This study aims at evaluating and comparing the effects of aging on two materials of high translucent zirconia by investigating the effects of artificial aging on surface roughness, phase transformation, transparency and biaxial flexural strength. The null hypothesis is that there is no difference between the two materials.

## Materials and methods

### Preparation of test specimens

The materials studied were DD cubeX^2^ (Dental Direkt Gmbh, Spenge, Germany) and Prettau Anterior (Zirkonzahn GmbH, Gais, Italy). The composition of the two materials is presented in Table 1. Forty zirconia discs (13 mm diameter) were CAD/CAM manufactured from prefabricated blanks. The mean thickness of DD cubeX^2^ discs was ∼1.1 mm (*n* = 20) and Prettau Anterior was ∼1.2 mm (*n* = 20). The discs were sintered according to the recommendations from the manufacturers, see Table 2.

**Table 1.** The chemical composition of the tested materials.

Product name	Chemical composition^a^
Prettau Anterior	<12% Y_2_O_3_, <1% Al_2_O_3_, max 0.02% SiO_2_, max 0.02% Fe_2_O_3_
DD CubeX^2^	<10% Y_2_O_3,_ <0.1% Al_2_O_3_, <1.0 other oxides

^a^According to manufacturer information.

**Table 2.** Sintering parameters for DD cubeX^2^ and Prettau Anterior.

	Temp. 1 (°C)	Temp. 2 (°C)	Heating rate (°C/min)	Dwell time (min)	Time (min)
DD cubeX^2^					
Heating	20	900	8		110
Dwell	900	900		30	30
Heating	900	1450	3		165
Dwell	1450	1450		120	120
Cooling	1450	200	10		125
Prettau Anterior					
Heating	20	1500	8		180
Dwelling				120	
Cooling	1500	50	8		180

After sintering, disc manufacturing defects were counted, documented and edge defects were carefully adjusted using a polishing rotation instrument (94020 F 040, Komet Dental, Lemgo, Germany) and a dental hand piece (Ti-Max Ti25L, Nakanishi Inc., Tokyo, Japan). Polishing was made in short bursts, at maximum speed with maximum water cooling to adjust defects. The diameter of the adjusted discs was measured to ensure that the dimensions were unaltered.

Specimen holders for polishing were manufactured using a silicon putty material (Provil novo, Heraeus Kulzer GmbH, Hanau, Germany). Polishing was made based on ISO standard SS-EN ISO 6872:2015 using a Struers LaboPol-1 & Struers LaboForce-1 (Struers Aps, Ballerup, Denmark) with a force of 50 N on Waterproof Silicon Carbide Paper FEPA #500 (30 μm) (Struers Aps, Ballerup, Denmark) for 5 min on each side. This was followed by a finer polishing on Waterproof Silicon Carbide Paper FEPA #1200 (15 μm, Struers Aps, Ballerup, Denmark) for 5 min on each side. Ten discs from each zirconia product were picked at random for aging and put in an ultrasonic cleaner (Sonorex Digitec, Bandelin electronic GmbH & Co KG) for 5 min in room temperature tap water.

### Aging

**Figure 1.**
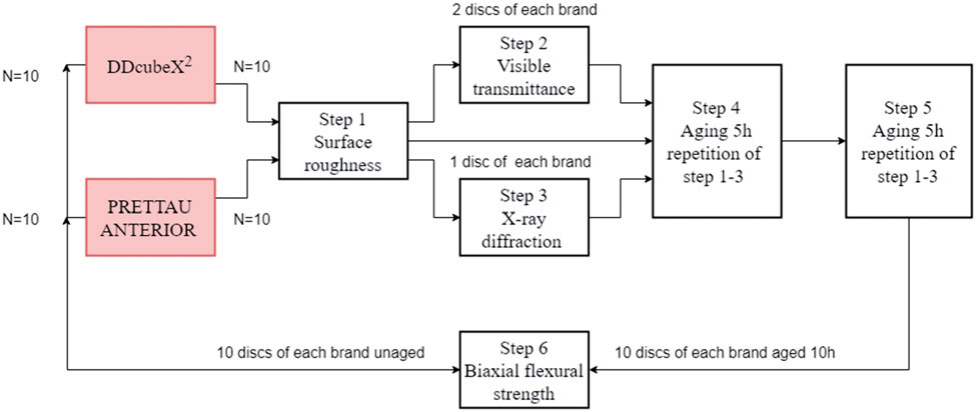
A flowchart explaining the workflow of the experiments.

Aging was preformed using an autoclave (GE 446, Getinge AB, Getinge, Sweden) for 5 h at 2 bars and 134 °C according to SS-EN ISO 13356:2015 [8]. After surface roughness, visible transmittance and X-ray diffraction (XRD) measurements, aging was repeated resulting in a total aging time of 10 h. For an illustration of the workflow see Figure 1.

### Surface roughness

Surface roughness measurements were performed using a contact profilometer (Precision Form Talysurf 50, Taylor Hobson, Berwyn, USA). Before each group of measurements, the profilometer was calibrated to a 6.07 μm reference specimen. Each side was scanned three times at three randomly selected locations and arithmetic average (Ra-value) was registered. Measurements were made on the 10 discs from each zirconia product intended for aging, first unaged and then repeated after 5 h and 10 h of artificial aging.

### Visible transmittance

A visible transmittance (300–700 nm) measurement was performed with a UV/Vis spectrophotometer (Lambda 35, PerkinElmer, Waltham, USA) at room temperature on 2 unaged discs from each zirconia product. The beam size is 0.5 × 7.5 mm. Measurements were repeated after 5 and 10 h of artificial aging. For comparison a disc (IPS Empress, Ivoclar Vivadent AG, Shann, Lichtenstein) with similar dimensions to the zirconia discs were used for reference. Data is presented in transparency percent.

### X-ray diffraction

X-ray diffraction was used to characterize the crystal structure of the aged and unaged zirconia in the present study. Polycrystalline X-ray diffraction analysis was performed on one unaged sample of each zirconia product and was repeated after 5 and 10 h of artificial aging. Continuous scans were performed in 2*θ* mode on a rotating sample in a power X-ray diffractometer (Bruker AXS D8 Advance, Bruker AXS GmbH, Karlruhe, Germany) with Cu K-α radiation and a Våntec-1 detector. Diffractograms were evaluated in Diffrac. EVA 4.2 with PDF-2 database to identify the crystal structures. Rietveld refinement was performed with software (TOPAS 4.2, Bruker AXS GmbH, Karlruhe, Germany) using structures from ICSD Web.

### Biaxial flexural strength

Biaxial flexure tests (piston-on-three-ball test) were performed according to SS-EN ISO 6872:2015 [9] using a universal mechanical testing machine (HTE-5000N, Tinius Olsen, Redhill, England). A total of forty discs were tested, twenty discs from each zirconia product, where half of the discs were unaged and the other half was aged for a total of ten hours. The crosshead speed was 1.5 mm/min with a load range of 50–1000 N. Maximum center tensile strength in mega pascal was calculated using the formula:

(1) $$\sigma =–0.2387\frac{P\;(X-Y)}{b^{2}}$$

where *σ* is the maximum center tensile strength in mega pascals, *p* is the total load causing fracture in newtons, and *b* is the specimen thickness at fracture origin in millimeters, and *X* and *Y* are expressed in Equation (2) and (3).

(2) $$X=(1+\nu )\;\text{ln}\;{\left(r_{2}/r_{3}\right)}^{2}+[(1-\nu )/2]{\left(r_{2}/r_{3}\right)}^{2}$$

(3) $$Y=(1+\nu )[1+\;\text{ln}\;{\left(r_{1}/r_{3}\right)}^{2}]+(1-\nu ){\left(r_{1}/r_{3}\right)}^{2}$$

where ν is Poisson’s ratio (0.25), *r_1_* is the radius of the support circle in millimeters, *r_2_* is the radius of the loaded area in millimeters, and *r_3_* is the radius of the specimen in millimeters.

### Statistical analysis

All data was analyzed using Statistical Package for the Social Sciences (SPSS). Statistical analysis of the collected data was performed with independent-sample Mann–Whitney *U* tests and Kruskal–Wallis tests. A *p*-value less than .05 (*p* < .05) was considered statistically significant. The Pearson correlation test (*p* < .05*)* was used to correlate the determined biaxial flexural strength with the surface roughness (Ra).

## Results

### Surface roughness

**Figure 2.**
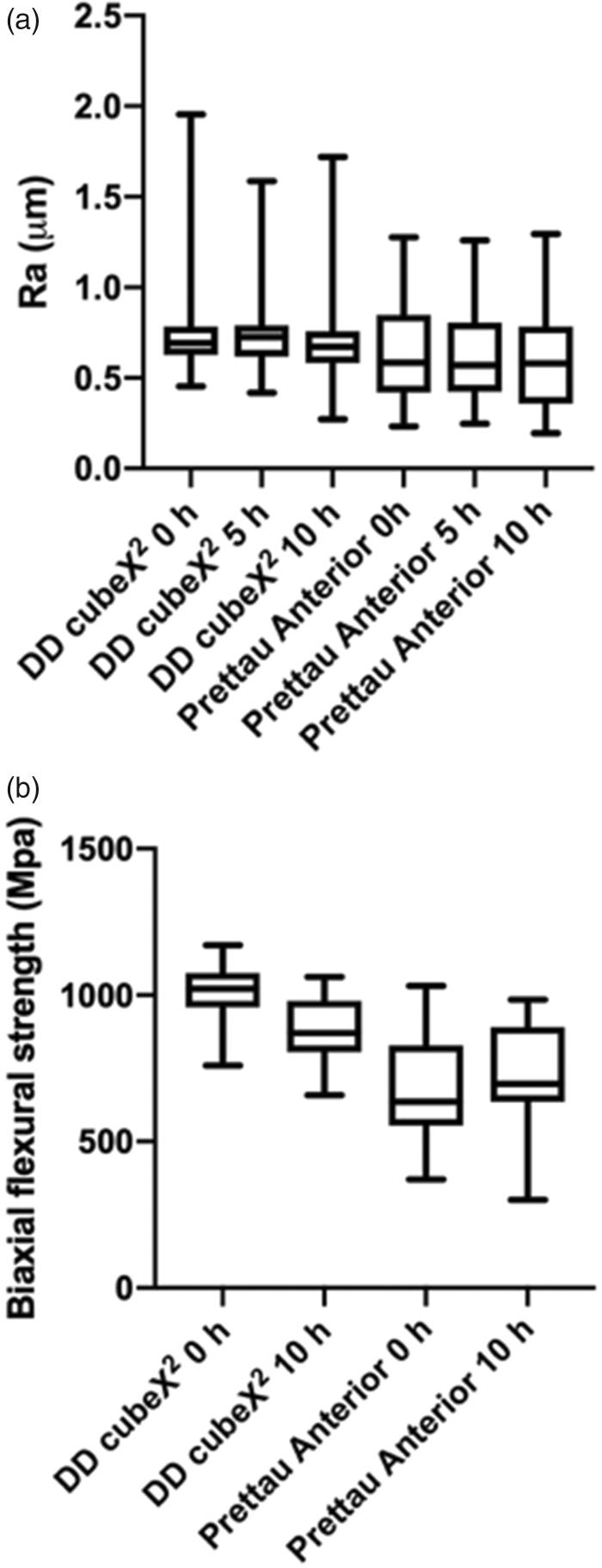
(a) Boxplot and whiskers displaying surface roughness presented in Ra value in μm for Prettau Anterior and DD cubeX^2^, unaged (0 h), aged 5 h (5 h), aged 10 h (10 h). (b) Boxplot and whiskers displaying biaxial flexural strength in MPa as unaged (0 h), aged 10 h (10 h).

DD cubeX^2^ had a significantly higher mean and median Ra-value both for unaged and aged for 5 h and 10 h, respectively, compared with Prettau Anterior (Figure 2(a)). The Ra-values of DD cubeX^2^ were more scattered compared with Prettau Anterior. Aging for 5 and 10 h, respectively, had no statistically significant effect on surface roughness for either product (*p* > .05).

### Visible transmittance

**Figure 3.**
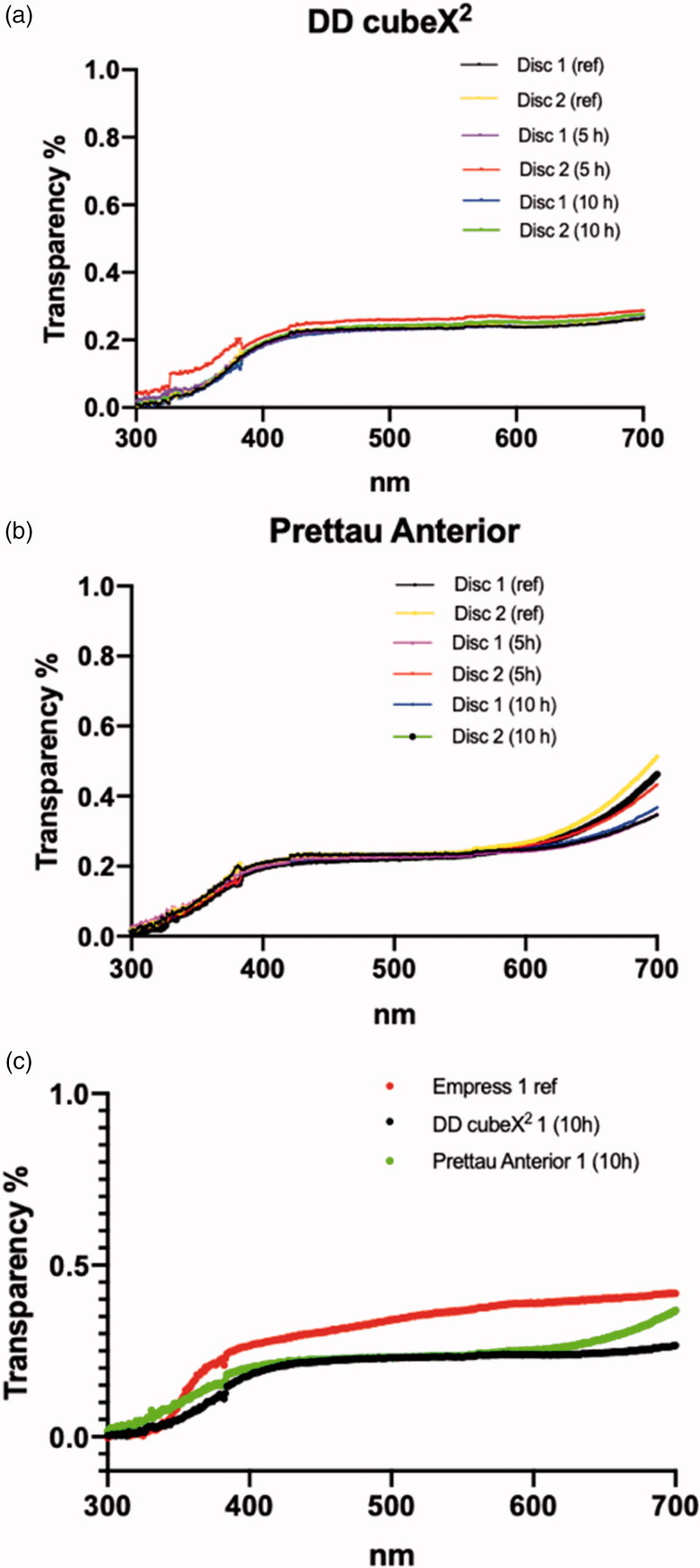
Level of transparency of DD cubeX^2^ (a) and Prettau Anterior (b) before aging (ref), after 5 h of aging (5 h) and after 10 h of aging (10 h) are presented. Two discs (disc 1 and disc 2) from each manufacturer are tested. (c) Level of transparency of DD cubeX^2^ aged for 10 h, Prettau Anterior aged for 10 h and Empress are presented (one sample each).

Aging had no significant effect on transparency for DD cubeX^2^ and Prettau Anterior at the visible light wavelengths 450, 550 and 650 nm for unaged discs and aged 5 and 10 h respectively (*p* > .05) (Figure 3(a,b)). Comparing all Prettau Anterior samples with all DD cubeX^2^ samples no significant difference in transparency for the light wavelengths 450 and 550 nm, respectively, was found (*p* > .05). At a wavelength of 650 nm Prettau Anterior was significantly more transparent than DD cubeX^2^ (*p* < .05). The reference discs made of an unaged translucent leucite glass-ceramic were significantly more translucent at 450, 550 and 650 nm (*p* < .05) compared with both types of zirconia, unaged and aged after 5 and 10 h (Figure 3(c)).

### X-ray diffraction

**Figure 4.**
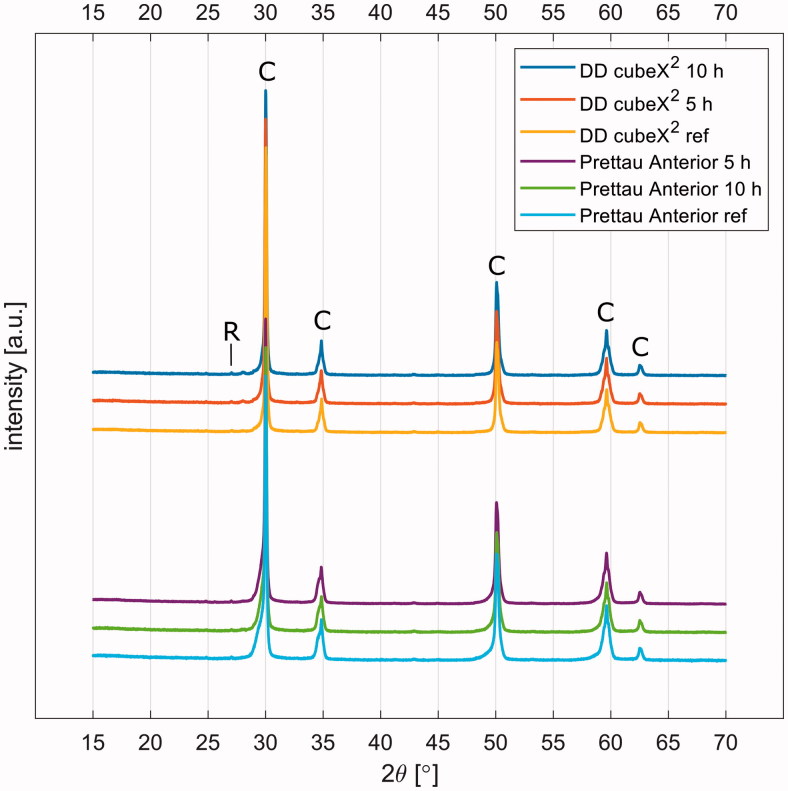
Diffractograms for the tested zirconia. These patterns revealed that both DD cubeX^2^ and Prettau Anterior have the characteristic pattern of yttria-stabilized tetragonal/cubic zirconia and the peaks are denoted with C. The contribution of TiO is visible at a small peak at 27°, signs of monoclinic ZrO^2^ are seen as small peaks at 28° for aged DD cubeX^2^, and this area is denoted with R.

Phase transformation analysis results are shown in Figure 4. The linear diffractograms for DD cubeX^2^ and Prettau Anterior, before aging (0 h), after artificial aging of 5 h (5 h) and 10 h (10 h) are shown. The produced patterns for both unaged and aged samples Prettau Anterior and DD cubeX^2^ can be explained to 99% by cubic or tetragonal zirconia, possibly with the presence of yttria. Both aged DD cubeX^2^ samples produced a small peak around 28 degrees that could belong to the (100) peak of monoclinic zirconia, but quantification gives less than one percent. For both aged and unaged Prettau Anterior samples there are small ‘shoulders’ on the peaks at 30° and 35° and the other peaks have a small broadening towards lower angles. These indicate a distribution of the unit cell size rather than one fixed value for the whole material. The sample holders contain small amounts of TiO_2_ and the samples did not completely cover the whole holder. This gives rise to a small contribution of rutile in the diffractograms, with its strongest peak at about 27°, seen in all samples.

### Flexural strength

The results for flexural strength are shown in Figure 2(b). Unaged DD cubeX^2^ had a mean flexural strength of 998 MPa while unaged Prettau Anterior had a mean flexural strength of 678 MPa. Unaged DD cubeX^2^ had a significantly stronger flexural strength compared with Prettau Anterior (*p* < .05). After 10 h of aging DD cubeX^2^ had a mean flexural strength of 878 MPa and Prettau Anterior 678 MPa, and the difference compared with unaged was not statistically significant (*p* > .05). Before aging, Prettau Anterior exhibited a wider range (370–1032 Mpa) of flexural strength compared with DD cubeX^2^ (759–1170 Mpa). After aging for 10 h, Prettau Anterior still exhibited a wider range (301–984 Mpa) of flexural strength compared with DD cubeX^2^ (658–1062 Mpa). The mean flexural strength of Prettau Anterior slightly increased after aging from 678 MPa to 723 MPa. However, the increase was not statistically significant (*p* > .05). Aging for 10 h significantly reduced the mean flexural strength of DD cubeX^2^ from 998 MPa to 878 MPa (*p* < .05). The Pearson correlation test revealed no statistically significant correlation (*p* > .05) between surface roughness and biaxial flexural strength.

## Discussion

The study aim was to investigate and compare two types of high translucent zirconia regarding the effects of aging on surface roughness, crystal structure, transparency and flexural strength to improve our knowledge about these materials. The null hypothesis that there are no differences between the two materials is rejected.

### Surface roughness

5Y-PSZ is often applied as monolithic restoration in the oral environment, which means without any veneer porcelain and that the antagonist tooth/restoration is in direct contact with 5Y-PSZ. It is therefore of great interest to *investigate* if artificial aging has any effect on the surface roughness. After artificial aging for 10 h, no significant increase in surface roughness was detected for either DD cubeX^2^ or Prettau Anterior (Figure 2(a)). Although DD cubeX^2^ had a significantly rougher mean surface roughness compared with Prettau Anterior (*p* < .05), still, the mean of biaxial flexural strength of DD cubeX^2^ is significantly higher than Prettau Anterior (*p* < .05). It seems that surface roughness has low correlation with the biaxial flexural strength of the tested materials. This lack of correlation could also be confirmed from the results from several earlier articles [10,11]. This might mean that not only surface roughness but also other factors could influence the flexural strength, therefore more studies are needed.

### Transparency

Since 5Y-PSZ is also said to be highly translucent, transparency before and after artificial aging was also tested. Our study showed that artificial aging for 10 h had no significant effect on the transparency of the tested zirconia materials (Figure 3). These results contradict with those of Walczak et al. [12] where aging translucent zirconia with a thickness of 0.5 mm for 5 h significantly reduced the translucency. Untreated leucite reinforced glass-ceramic was more transparent than DD cubeX^2^ and Prettau Anterior, thus porcelain veneering might be the best alternative when esthetics is of utmost importance. According to Zhang [13] all commercial 3Y-TZP products from major manufacturers remain essentially opaque when their thicknesses approach 1 mm. A disadvantage with high translucent restorations is that underlying structures can be noticed through the material, revealing discolored dentine and prosthetic posts. This is a clinical indication where today’s high translucent zirconia may excel.

### Flexural strength

The reported flexural strength from the manufacturer for DD cubeX^2^ is >720 MPa and >670 MPa for Prettau Anterior. The results of this study showed that the DD cubeX^2^ discs before aging ranged from 759 to 1170 MPa, all of them above the reported value from the manufacturer. For Prettau Anterior, however, the obtained flexural strength before aging has a range from 370 to 1032 MPa, which means that some discs had a much lower flexural strength than the value presented from the manufacturer. Commercial 3Y-TZP has a reported flexural strength of more than 1000 MPa [2]. This result is in agreement with the study by Kwon et al. [1], where they found that high translucent zirconia has a lower flexural strength than 3Y-TZP. The range of flexural strength for DD cubeX^2^ is smaller (759–1170 MPa) and Prettau Anterior is larger (370–1032 MPa). A wider range of measured flexural strength could indicate that Prettau Anterior is a less stable material than DD cubeX^2^, which also means that the flexural strength of DD cubeX^2^ is more predictable. The flexural strength reportedly could decrease if the sintering temperature is above 1600 °C [14]. Sintering temperature between 1400 °C and 1550 °C will produce zirconia with the highest flexural strength [15]. In the present study, the highest sintering temperature for DD cubeX^2^ was at 1450 °C and 1500 °C for Prettau Anterior, which are in the range of recommended sintering temperature for this kind of zirconia materials. Nevertheless, as the tested materials have a slight difference in the composition (Table 1) and the sintering regime (Table 2), these are also parameters that might have impact on the flexural strength of the materials. It should be noted that the present study showed that aging significantly reduces flexural strength of the DD cubeX^2^ samples. The change is not reflected by an increased amount of monoclinic zirconia according to X-ray diffraction. Artificial aging may affect other factors in the material or not at all. There is a risk that the significant difference is a result of the wide range of flexural strength measured for each zirconia product. To further investigate and verify the results larger samples are needed. The loading condition in the oral environment is rather complicated; load to failure testing as performed in the present study might only have small clinical relevance. Another limitation with this study is that no fatigue testing of the materials was made. Fatigue resistance is vital for a material subdued to repeated loading, as it may weaken the material until it breaks. The strength of a ceramic may decrease by a factor of 2 to 3 during its lifetime from repeated cyclic loading [2]. In the future, fatigue strength test might also be considered.

### Aging

The diffractogram patterns presented in the present study showed that the produced patterns for both unaged and aged samples Prettau Anterior and DD cubeX^2^ can be explained to 99% by cubic or tetragonal zirconia. However, the tetragonal zirconia and the cubic zirconia could not be separated with current setup, since their patterns from diffractogram are close to identical [16]. After 10 h artificial aging at 134 °C and 2 bars, less than 1% monoclinic zirconia content could be found both in DD cubeX^2^ and in Prettau Anterior. The information provided from a previous review article by Zhang & Lawn stated that the volume percentage of the cubic phase of both tested zirconia materials were above 50% [2]. Since cubic zirconia does not undergo a transformation, zirconia with higher cubic phase content is less susceptible to aging [2]. This might be one of the reasons there were less than 1% monoclinic zirconia present in the tested DD cubeX^2^ and Prettau Anterior after 10 h of artificial aging. Since the detected peak of monoclinc phase is extremely low (Figure 4), it might also be considered as noise which produced by the XRD. Surface flaws on the edge of the discs might be located as stress concentration areas and might lead to earlier disc fractures. Zirconia is a multi-phased material, which means adjusting the surface risks introducing phase transformation. Therefore, minor adjustments were performed carefully under maximum water coolant in the present study. Hence, no or only small amounts of monoclinic phase were present after artificial aging for 10 h; minor adjustments might also be accepted in the clinical situation.

Ten hours of accelerated aging at 134 °C and 2 bars is estimated to correspond to 30–40 years in vivo [5]. Both materials were resistant to accelerated aging, however, it might be other factors that affect the zirconia-disc durability. Longer hydrothermal aging of the discs is possible, but as 30–40 years is a long survival time for a prosthetic restoration, further hydrothermal aging without changing other factors might not be of clinical relevance. To attain stronger evidence of the effects of aging on high translucent zirconia a study could be made where the aging conditions more closely match the oral cavity including factors such as occlusal loading and saliva.

## Conclusions

Within the limitation of the present *in vitro* study, both DD cubeX^2^ and Prettau Anterior seem to be relatively aging resistant. However, a wider range of measured flexural strength indicated that Prettau Anterior probably is a less stable material than DD cubeX^2^, which also means that the flexural strength of DD cubeX^2^ could be more predictable.

## References

[1] Kwon SJ, Lawson NC, McLaren EE, et al. Comparison of the mechanical properties of translucent zirconia and lithium disilicate. J Prosthet Dent. 2018;120(1):132–137. 29310875 10.1016/j.prosdent.2017.08.004

[2] Zhang Y, Lawn BR Novel zirconia materials in dentistry. J Dent Res. 2018;97(2):140–147. 29035694 10.1177/0022034517737483 PMC5784474

[3] Lughi V, Sergo V Low temperature degradation -aging- of zirconia: a critical review of the relevant aspects in dentistry. Dent Mater. 2010;26(8):807–820. 20537701 10.1016/j.dental.2010.04.006

[4] Deville S, Chevalier J, Gremillard L Influence of surface finish and residual stresses on the ageing sensitivity of biomedical grade zirconia. Biomaterials. 2006;27(10):2186–2192. 16332348 10.1016/j.biomaterials.2005.11.021

[5] Deville S, Gremillard L, Chevalier J, et al. A critical comparison of methods for the determination of the aging sensitivity in biomedical grade yttria-stabilized zirconia. J Biomed Mater Res. 2005;72(2):239–245. 10.1002/jbm.b.30123 15654702

[6] Inokoshi M, Zhang F, De Munck J, et al. Influence of sintering conditions on low-temperature degradation of dental zirconia. Dent Mater. 2014;30(6):669–678. 24698437 10.1016/j.dental.2014.03.005

[7] Marinis A, Aquilino SA, Lund PS, et al. Fracture toughness of yttria-stabilized zirconia sintered in conventional and microwave ovens. J Prosthet Dent. 2013;109(3):165–171. 23522365 10.1016/S0022-3913(13)60037-2

[8] International Organization for Standardization. Implants for surgery – Ceramic materials based on yttria-stabilized tetragonal zirconia (Y-TZP). SS-EN ISO 13356. 2015.

[9] International Organization for Standardization Dentistry – Ceramic materials. ISO 6872. 2015.

[10] André M, Kou W, Sjögren G, et al. Effects of pretreatments and hydrothermal aging on biaxial flexural strength of lithium di-silicate and Mg-PSZ ceramics. J Dent. 2016;55:25–32. 27638179 10.1016/j.jdent.2016.09.002

[11] Flury S, Peutzfeldt A, Lussi A Influence of surface roughness on mechanical properties of two computer-aided design/computer-aided manufacturing (CAD/CAM) ceramic materials. Oper Dent. 2012;37(6):617–624. 22616923 10.2341/11-391-L

[12] Walczak K, Meißner H, Range U, et al. Translucency of zirconia ceramics before and after artificial aging. J Prosthodont. 2018;28(1):e319–e324. 29527778 10.1111/jopr.12771

[13] Zhang Y Making yttria-stabilized tetragonal zirconia translucent. Dent Mater. 2014;30(10):1195–1203. 25193781 10.1016/j.dental.2014.08.375 PMC4167579

[14] Nassary Zadeh P, Lümkemann N, Sener B, et al. Flexural strength, fracture toughness, and translucency of cubic/tetragonal zirconia materials. J Prosthet Dent. 2018;120(6):948–954. 29807742 10.1016/j.prosdent.2017.12.021

[15] Stawarczyk B, Özcan M, Hallmann L, et al. The effect of zirconia sintering temperature on flexural strength, grain size, and contrast ratio. Clin Oral Invest. 2013;17(1):269–274. 10.1007/s00784-012-0692-6 22358379

[16] Igawa N, Ishii Y Crystal Structure of Metastable Tetragonal Zirconia up to 1473 K. J Am Ceram Soc. 2001;84(5):1169–1171.

